# Gene expression profiling of Naïve sheep genetically resistant and susceptible to gastrointestinal nematodes

**DOI:** 10.1186/1471-2164-7-42

**Published:** 2006-03-06

**Authors:** Orla M Keane, Amonida Zadissa, Theresa Wilson, Dianne L Hyndman, Gordon J Greer, David B Baird, Alan F McCulloch, Allan M Crawford, John C McEwan

**Affiliations:** 1 AgResearch Molecular Biology Unit, Department of Biochemistry, University of Otago, Dunedin, New Zealand; 2 Department of Biochemistry, University of Otago, Dunedin, New Zealand; 3 AgResearch Invermay Agricultural Centre, Mosgiel, New Zealand; 4 AgResearch, Lincoln, New Zealand

## Abstract

**Background:**

Gastrointestinal nematodes constitute a major cause of morbidity and mortality in grazing ruminants. Individual animals or breeds, however, are known to differ in their resistance to infection. Gene expression profiling allows us to examine large numbers of transcripts simultaneously in order to identify those transcripts that contribute to an animal's susceptibility or resistance.

**Results:**

With the goal of identifying genes with a differential pattern of expression between sheep genetically resistant and susceptible to gastrointestinal nematodes, a 20,000 spot ovine cDNA microarray was constructed. This array was used to interrogate the expression of 9,238 known genes in duodenum tissue of four resistant and four susceptible female lambs. Naïve animals were used in order to look at genes that were differentially expressed in the absence of infection with gastrointestinal nematodes. Forty one unique known genes were identified that were differentially expressed between the resistant and susceptible animals. Northern blotting of a selection of the genes confirmed differential expression. The differentially expressed genes had a variety of functions, although many genes relating to the stress response and response to stimulus were more highly expressed in the susceptible animals.

**Conclusion:**

We have constructed the first reported ovine microarray and used this array to examine gene expression in lambs genetically resistant and susceptible to gastrointestinal nematode infection. This study indicates that susceptible animals appear to be generating a hyper-sensitive immune response to non-nematode challenges. The gastrointestinal tract of susceptible animals is therefore under stress and compromised even in the absence of gastrointestinal nematodes. These factors may contribute to the genetic susceptibility of these animals.

## Background

Grazing ruminants are constantly exposed to natural challenge by gastrointestinal nematodes. Infection by such parasites leads to clinical disease and production losses and is a serious problem in animal husbandry. The economically important gastrointestinal parasites of sheep belong to the Order Strongylida and the family Trichostrongyloidea and include *Teladorsagia *(*Ostertagia*), *Trichostrongylus*, *Nematodirus *and *Haemonchus *spp. The host response to parasite challenge is complex and poorly understood due to its polygenic nature. The response varies from sheep breed to sheep breed and from animal to animal [1]. The response can result in rapid or delayed expulsion of parasites and the host response is critical for determining subsequent parasite status [2]. Resistance to nematodes is primarily due to acquired immunity and is largely a Th2 type response [3-8] although innate immunity is also thought to play a role [9]. Sheep lines which have been selected to be resistant to one particular nematode species have been shown to have increased resistance to other nematode species [10-13] primarily due to a non-specific mechanism of parasite expulsion [2]. Historically anthelmintic drenching has been used to control nematode infection, however, the efficacy of this treatment is decreasing as parasite resistance to anthelmintics increases and nematodes resistant to multiple classes of anthlemintics are now found worldwide [14]. The use of chemicals in food production is also increasingly encountering public hostility [15].

An alternative method of helminth control is vaccination of host animals. This strategy has successfully combated many bacterial and viral diseases, however, despite extensive research there is currently no vaccine available against the major gastrointestinal parasitic nematode species [15-17]. This is most likely due to the number of nematode species infecting the host and the complex parasite life cycle which expresses different antigens at each stage.

A natural method of parasite control is breeding for host resistance. Resistance to internal parasites, as measured by faecal egg count (FEC), has moderate heritability (h^2 ^= 0.23–0.41, [1]) and this method has been shown to make significant genetic gains in a variety of sheep breeds [18-24] due to the combination of reduced FEC and reduced pasture contamination. The current method of animal selection is cumbersome and inefficient, however, as animals are selected based on their phenotype. Genetic gain could be accelerated if animals were selected on genotype rather than phenotype.

Lines of Perendale sheep have been divergently selected for parasite resistance and susceptibility at AgResearch, New Zealand since 1986 and differ in FEC by 4.9 fold [24]. The number of adult nematodes in the gastrointestinal tract of animals from the resistant line is also lower than that from the susceptible line [25]. Resistant animals therefore have an increased genetic capability to respond to and subsequently reject parasites when challenged. The identification of genes involved in this process would allow the development of genetic markers, which could be used in marker assisted selection breeding programs. A previous study identified genes differentially expressed between the selection lines in response to parasite challenge, but the use of challenged animals confounded the resistance status of the animals with level of infection. This study also did not distinguish innate from acquired immunity [25]. We therefore chose to look at those genes that were differentially expressed between the selection lines regardless of the level of infection by using naïve animals that had never been exposed to gastrointestinal nematodes. We did this using high-throughput DNA microarray technology. This allowed us to examine the relative expression of thousands of genes in a single experiment and to group genes into expression classes, providing insight into their biological function.

## Results

**Figure 1:**
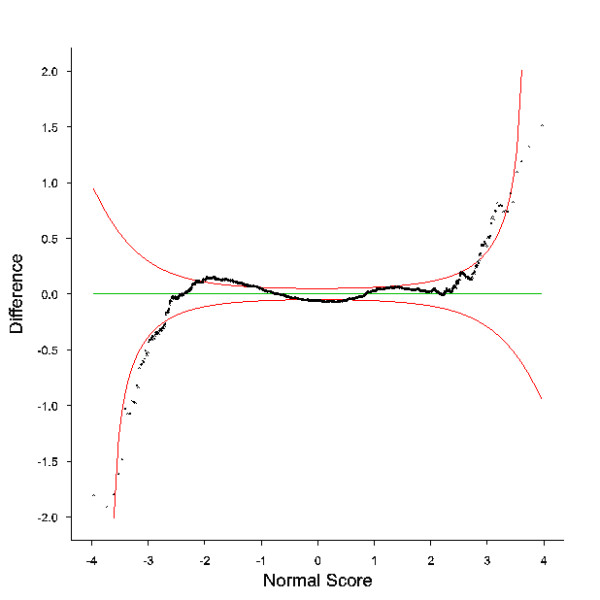
**Differenced Normal Q-Q plot for the modified T value of the log ratio of the mean**. The expected normal deviate (normal score) is plotted against the difference between the observed order statistic and the expected normal order statistic (95% confidence limits shown in red). Data is combined from all 16 slides of the experiment. ESTs more highly expressed in resistant animals are shown on the *right *while ESTs more highly expressed in susceptible animals are shown on the *left*.

The microarray experiment examined gene expression in duodenum tissue from four resistant and four susceptible naïve Perendale lambs. Poly(A)^+ ^RNA isolated from duodenum tissue was reverse transcribed into cDNA, fluorescently labelled and hybridised to ovine 20 k cDNA microarrays. The experiment design was a factorial dye swap design involving 16 slides, where every animal was compared to every animal in the opposite selection line, as described previously [25]. One hundred ESTs showed differential expression (Figure 1) between the resistant and susceptible animals. Forty ESTs were more highly expressed in the resistant line while 60 were more highly expressed in the susceptible line. These ESTs were resequenced in order to verify the insert sequence. Confirmed ESTs were annotated using BLASTN against the human RefSeq database and a cut-off E value of 1 × 10^-18^. Thirty of the ESTs more highly expressed in susceptible animals could be verified and annotated in this manner giving 25 unique human RefSeqs. Sixteen of the ESTs more highly expressed in resistant animals could also be annotated. The lists of differentially expressed human RefSeqs is given in Tables 1 and 2.

**Figure 2:**
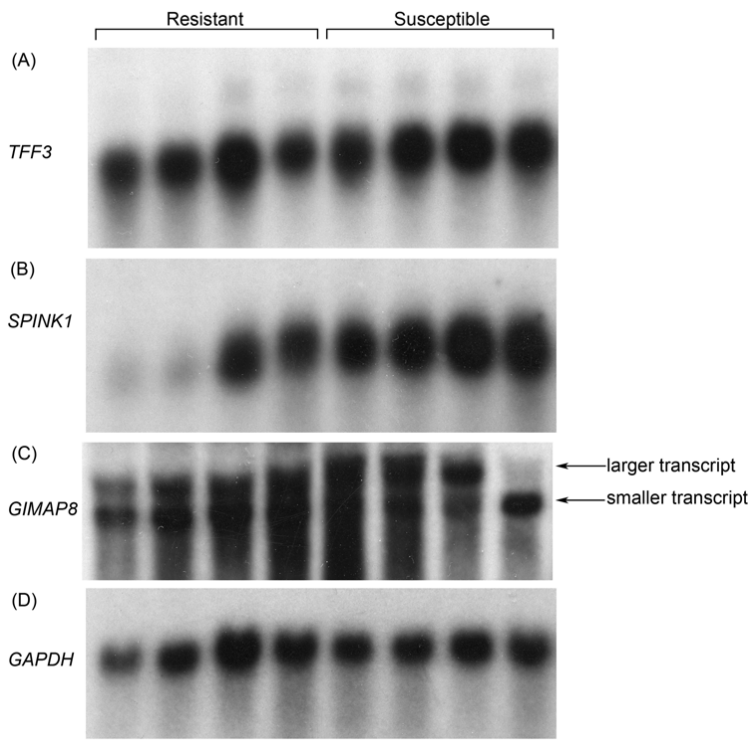
**Northern blotting**. Northern blots showing differential expression of *TFF3 *(A), *SPINK1 *(B), and *GIMAP8 *(C). mRNA levels were normalised to that of *GAPDH *(D).

Northern blotting was carried out to confirm differential expression of a number of genes (Figure 2). *TFF3 *encodes intestinal trefoil factor 3. This gene is a member of a family of trefoil factors that is involved in mucosal maintenance and repair and is known to be overexpressed during inflammatory processes [26]. In accordance with the microarray results, this gene had a 20% increase in expression in the duodenum of the susceptible animals compared to the resistant (Figure 2A). While the degree of upregulation was not marked it was still significant (P = 0.05).

Differential expression of the pancreatic secretory trypsin inhibitor gene (*SPINK1*) was also confirmed. *SPINK1 *encodes a gene also known as *TATI *(tumour associated trypsin inhibitor) that is expressed mainly in the pancreas but is also expressed in the mucosa of the small intestine [27]. One of the major roles of SPINK1 is thought to be the prevention of premature activation of pancreatic proteases. This has the effect of decreasing the rate of mucus digestion by luminal proteases within the stomach and colon. SPINK1 is also known to increase the proliferation of a variety of cell lines and to stimulate cell migration, implying that it may be involved in the healing response following injury [28]. When measured by Northern blot, *SPINK1 *expression was increased two fold in the susceptible animals compared to the resistant animals (Figure 2B) and this increased expression was highly significant (P = 0.005). In particular two of the resistant animals had very low levels of *SPINK1 *expression although expression in the other two resistant animals was still lower than expression in any of the susceptible animals.

Differential expression of the immunity associated GTPase, *GIMAP8*, was also confirmed. This gene is a member of a novel family of GTPases conserved among higher plants and vertebrates [29]. Human GIMAP proteins are known to be expressed most highly in the spleen and lymph nodes, but expression has also been detected in the digestive tract [30]. GIMAP proteins are thought to be involved in the control of cell survival and response to infection and GIMAP8 has been shown to have anti-apoptotic functions. Expression of this gene is known to be decreased in the spleen of mice infected with the protozoan parasite *Plasmodium chabaudi *[29]. *GIMAP8 *was expressed 2.6 fold more highly in the intestine of resistant animals compared to susceptible (Figure 2C) as measured by Northern blot. This differential expression is also highly significant (P = 0.0018). It is notable that the fold increase in expression of both *GIMAP8 *and *SPINK1 *is markedly higher when quantitated by Northern blot rather than when quantitated by microarray. Differential transcript expression of *GIMAP8 *was also observed with the four resistant animals predominantly expressing an approximately 3,300 bp transcript, although a larger transcript of approximately 4,100 bp could still be detected. In contrast three of the susceptible animals predominantly expressed the larger transcript although the smaller transcript was detectable, while one susceptible animal expressed the smaller transcript. The precise size of the *GIMAP8 *transcript is unknown in sheep. Transcript variants of this gene, with sizes of 4,700 bp and 4,200 bp, have been previously reported in human and transcript variants of size 4,200 bp and 2,900 bp have been reported in mouse [29,30].

Gene Ontology (GO) terms significantly associated with the differentially expressed genes were found using EASEonline [31] and are listed in Table 3. A number of GO terms were significantly associated (P < 0.05) with the genes more highly expressed in the intestine of susceptible animals. These terms pertain to an organism's response to stress and stimulus and imply that despite the absence of nematodes from the environment of the susceptible animals, their gastrointestinal tract is still responding to insults or injuries, possibly by other pathogens. The only GO term significantly associated with genes more highly expressed in resistant animals was "cellular process" indicating that the intestine of these animals does not appear to be under stress.

**Figure 3:**
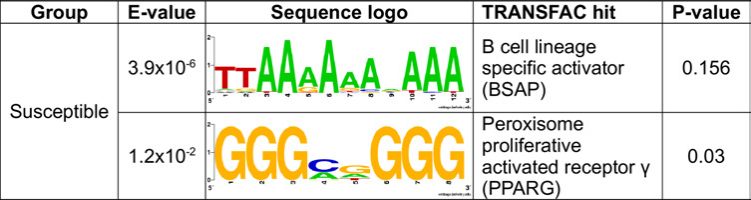
**Significant motifs detected in the promoter regions of the differentially expressed genes**. The significant motifs found in the promoters of the differentially expressed genes are given along with their sequence logos and top TRANSFAC hit.

Promoter regions of the 16 genes more highly expressed in resistant animals and the 25 genes more highly expressed in susceptible animals were analysed for common *cis*-regulatory motifs. The analysis was performed using the MEME motif prediction program [32]. Only two motifs were significant and both were found in the promoter regions of the genes more highly expressed in susceptible animals. The sequence logo for these motifs is shown in Figure 3. MAST analysis indicated that despite individual motifs not being significant, the combination of motifs identified in promoter regions of both groups were unique to the group in which they were discovered as they were not significantly associated with the opposing group or with all the genes on the array by Fisher's exact test (Table 4). A separate MAST analysis indicated that the motifs identified in this study were also not significantly associated (P > 0.05) with genes previously reported to be differentially expressed in sheep exposed to gastrointestinal nematodes [25] (data not shown).

In order to identify which transcription factors could bind to the motifs, each motif was compared to the transcription factor binding sites in the TRANSFAC database [33]. Each motif was shuffled 1,000 times and the comparison process repeated in order to establish its significance. Figure 3 lists the significant motifs discovered along with their best TRANSFAC hits. The full list of identified motifs and their TRANSFAC hits are available in Additional files 1 and 2. The two significant motifs found in the promoter regions of the genes more highly expressed in susceptible animals give B cell lineage specific activator protein (BSAP) and Peroxisome proliferative activated receptor gamma (PPARG) as their best hit. BSAP, also known as PAX5 is a transcription factor required for B cell development [34], however, it should be noted that the P value of this hit is not significant and so this motif may be bound by a transcription factor not represented in the TRANSFAC dataset. Interestingly, five of the six genes associated with the GO term "response to biotic stimulus" have this motif in their promoter regions, indicating this motif may be involved in the regulation of this response. Peroxisome proliferative activated receptor gamma (PPARG) is a ligand activated transcription factor which has an important role in adipocyte differentiation [35]. PPARG is known to have anti-inflammatory effects and to play an important role in the maintenance of mucosal integrity in the intestine [36]. This transcription factor may therefore play a role in co-ordinately regulating genes more highly expressed in the intestine of the susceptible animals.

## Discussion

This study identified a number of genes differentially expressed between lines of lambs differing genetically in their ability to become resistant to gastrointestinal nematodes. These genes were differentially expressed in the absence of nematode challenge and so represent basal expression differences between the selection lines. It is noteworthy that significant differences were observed in naïve animals as much previous work has shown that animals differing in host resistance primarily differ in the rapidity and strength of their acquired immune response rather than differing in their innate immunity [3,5,6]. To our knowledge this is the first report of constitutive differences in gene expression in naïve sheep differing in their genetic ability to respond to host infection.

Gene Ontology terms associated with the genes more highly expressed in susceptible animals included "response to stimulus", "response to stress", "defence response" and "response to pests, pathogens and parasites". This implies that the gastrointestinal tract of the susceptible animals is responding to stress even in the absence of nematode challenge. This is evidenced by the elevated expression of seven genes that are induced in response to stimulus, *HLA-A*, *MSH6*, *GPX2*, *IFI35*, *UBD*, *SERPING1 *and *TFF3*. *HLA-A *encodes an MHC class I heavy chain molecule that, in conjunction with B2M, presents endogenously derived peptides to CD8^+ ^cytotoxic T cells. *HLA-A *has broad tissue expression and has been shown to be induced by a number of cytokines, in response to infection and in patients with the inflammatory bowel syndrome Crohn's disease [37-39]. *MSH6 *encodes a MutS homologue protein which, in complex with MSH2, forms the MutSα heterodimer, while MSH2 in complex with MSH3 forms the MutSβ heterodimer. Both these complexes are involved in mismatch repair and repair mutagen-induced lesions in DNA as well as errors in DNA replication [40]. MutSα repairs both DNA mismatches and short 1–2 bp insertions or deletions (indels) while MutSβ repairs longer 2–6 bp indels [41]. A change in expression of either *MSH6 *or *MSH3 *can subsequently lead to a change in the MutSα to MutSβ ratio. The MutSα complex is induced at the transcriptional level in response to radiation [42]. Expression of *MSH6 *may be higher in the duodenum of susceptible animals in order to assist these animals to cope with toxic and mutagenic insults present in ingested food. *GPX2 *is another stress response gene more highly expressed in the intestine of the susceptible animals. This gene encodes the selenoprotein, gastrointestinal glutathione peroxidase 2, which catalyses the reduction of peroxides by reduced glutathione and protects the cell against oxidative damage. This gene is known to be induced in response to oxidative stress [43]. The interferon inducible gene *IFI35 *is induced at the transcriptional level in response to interferons, and complexes with the N-Myc interacting protein, NMI, to form a high molecular weight cytosolic complex [44]. The precise function of this complex, however, remains unknown. *UBD *encodes a small ubiquitin-like modifier protein also known as FAT10. *UBD *is induced by the cytokines IFNγ and TNFα [45] and can bind proteins and target them for degradation by the proteasome in a cytokine inducible, irreversible, ubiquitin-independent manner [46]. *SERPING1*, also known as C1 inhibitor (*C1-INH*), encodes an IFNγ inducible [47] serine protease inhibitor of the complement and contact systems. This protein therefore exhibits an anti-inflammatory effect [48,49]. SERPING1 is known to help prevent endotoxic shock [50] and is under investigation as a clinical treatment for a variety of diseases [51]. *TFF3 *encodes intestinal trefoil factor 3. Trefoil factors are involved in mucosal protection and healing and are induced during inflammation and in response to gastrointestinal mucosa damage [52]. The increased expression of so many stress response genes in the intestine of the susceptible animals appears to indicate that these animals are responding to gut insult and inflammation. Alternatively, these animals may constitutively express these stress response genes at inappropriate levels in the absence of challenge. Interestingly, stress response genes did not appear to be more highly expressed in the duodenum of susceptible animals after natural challenge with nematodes [25] indicating that these genes may be inappropriately regulated in the gastrointestinal tract of susceptible animals.

The source of stress to the intestinal tract of the susceptible animals remains unknown. Some of the stress response genes more highly expressed in susceptible animals are known to play a role in protecting the cell against oxidative damage. MSH6 is involved in repairing DNA lesions caused by oxidation [53] while GPX2 reduces DNA damaging agents [54]. HMOX1 is also strongly induced by oxidative stress and is involved in heme degradation [55]. Chronic gut inflammation is associated with enhanced production of leukocyte derived oxidants [56]. Therefore the gastrointestinal tract of the susceptible animals may be suffering damage from reactive oxygen species. This could come from a number of sources such as aberrant cellular metabolism or phagocytic leukocytes responding to an infection. Indeed some of the stress proteins more highly expressed in the gastrointestinal tract of the susceptible animals are involved in the immune response and are inducible by pro-inflammatory cytokines. Therefore although free from gastrointestinal nematodes, the susceptible animals may be responding to infection by other viral or bacterial pathogens.

A number of pro-apoptotic genes are more highly expressed in the intestine of the susceptible animals. These genes promote apoptosis in response to stress. Apoptosis is a primary form of defence against infection, stress, damage or injury in the cell. MSH6, TRAF4, UBD and BMF can all induce a protective apoptotic response in cells [45,57-59]. The soluable form of HLA-A has also been shown to trigger apoptosis of CD8^+ ^T cells [60]. Apoptosis can be induced directly by DNA damage or by cytokine signalling.

The genes more highly expressed in intestinal tissue of susceptible animals also included a number that encode proteins involved in protein degradation: these are *PSMD12*, which encodes a non-ATPase component of the 26S proteasome subunit, *UBD *which encodes a ubiquitin-like protein that targets proteins for degradation and *BMSC-UbP *which encodes a bone marrow stromal cell derived ubiquitin-like protein [46,61,62]. Another gene which can play a role in protein degradation is *SEC61B*, which encodes the beta subunit of the Sec61 protein translocator, which transports proteins across the endoplasmic reticulum. This complex is also known to play a role in retrograde transport of misfolded or degraded proteins back into the cytoplasm for proteasome degradation [63]. Therefore there appears to be increased protein degradation in the intestinal tract of the susceptible animals.

In summary, a number of stress response genes appear to be induced in the intestinal tract of naïve susceptible animals. How the induction of these genes is regulated is unclear at present, however, the identification of two significant motifs in the promoter regions of these genes indicated that transcription may be co-ordinately regulated by the BSAP or PPARG transcription factors. The role of PPARG in regulating genes involved in nematode susceptibility could potentially be examined by treating animals with PPARG agonists in order to modulate PPARG activation. Motifs for BSAP or PPARG binding were not found in the promoter region of genes whose expression was not elevated in the susceptible animals, showing they are unique to these genes.

Only 16 genes were more highly expressed in the intestine of the resistant animals than the susceptible animals. These genes had a variety of biological functions and the only GO term significantly associated with these genes was "cellular process". However two of genes more highly expressed in the resistant animals are vital for maintaining a functioning and healthy immune system. RAC2 plays an important role in response to pathogens as it regulates neutrophil chemotaxis and superoxide production and deficiency of RAC2 leads to impaired host defences and neutrophilia [64,65]. Deficiency of ITGB2, also known as CD18, can also lead to neutrophilia [66] and ITGB2 is also known to be important in innate immunity [67]. Two apoptosis inducing genes are more highly expressed in the intestine of the resistant animals. These genes are *DAP3 *and *TRADD*. DAP3, a proposed nucleotide binding protein, is a major positive regulator of apoptosis and has been shown to be critical for anoikis [68]. *TRADD *encodes a tumour necrosis factor receptor adaptor protein. This protein links the TNF receptor to the caspase pathway initiating apoptosis [69]. Despite a few genes with similar functions being more highly expressed in the intestine of resistant animals, no significant motifs were identified in the promoter regions of these genes indicating that they may not be co-ordinately regulated at a transcriptional level. Alternatively, the small number of genes with significantly elevated expression provided little power to detect motifs responsible for co-ordinate regulation.

A previous study identified genes differentially expressed between the resistant and susceptible lines in response to nematode challenge [25]. Interestingly, there were no genes that were consistently differentially expressed both pre and post-infection. However, the ubiquitin-like modifier gene, *UBD*, was more highly expressed in the susceptible animals prior to infection but more highly expressed in resistant animals after infection with gastrointestinal nematodes. *UBD *expression is induced by the cytokines IFNγ and TNFα. Expression of *TNFα *is known to be induced in intestinal lymph of genetically resistant Romney sheep during primary infection of naïve animals with *T. colubriformis *[4]. Therefore, while *UBD *is expressed more highly in naïve susceptible animals, upon infection with nematodes the resistant animals may induce *UBD *expression to a higher level than the susceptible animals in a TNFα-dependent manner. The previous study also identified smooth muscle function and Major Histocompatibility Complex II expression as important mediators of parasite resistance [25]. The current study did not identify smooth muscle or MHC II genes as differentially expressed between the lines in the absence of challenge and so shows that these genes are induced in response to challenge. Resistance to gastrointestinal nematodes may therefore be mediated, in part, by the ability to induce expression of these genes and this response is generated upon exposure to infection.

## Conclusion

Despite divergent selection over many years relatively few genes were differentially expressed between the selection lines in the absence of nematode challenge. Many more genes were found to be differentially expressed between the lines in response to natural parasite challenge [25] however, the genes identified in the present study may contribute to an animals innate resistance or susceptibility. The genes more highly expressed in resistant animals had a variety of functions, but some were involved in maintaining a healthy immune system, while some were pro-apoptotic genes. A number of genes more highly expressed in the susceptible animals were related to cellular response to stress and infection indicating that the susceptible animals may have a compromised gastrointestinal tract, even in the absence of nematode infection, and this may contribute to their innate susceptibility. It is notable that the human orthologs of the differentially expressed genes are located on a number of different human chromosomes (Tables 1 and 2). It is not known whether the polymorphisms that give rise to the differences in expression reported here are due to *cis *or *trans *mutations. Although it is possible that a mutation in one or a small number of genes may give rise to the expression differences, the diversity of the actions and pathways of the genes differentially expressed is consistent with genetic resistance to nematodes being due to many genes with small effects rather than a mutation in a single locus. The recent release of the bovine genome [70], a closely related species, will greatly aid mapping and resequencing of sheep genes responsible for parasite resistance.

## Methods

### Selection lines and tissue collection

Resistant and susceptible lines of Perendale sheep have been selected based on faecal egg count (FEC) since 1986. These lines now differ in faecal egg count by 4.9 fold [24]. Pregnant ewes were adjusted to concentrate feed, treated with anthelmintic, brought indoors and subsequently lambed indoors. The lambs were raised indoors to insure their immune system remained naïve with respect to gastrointestinal nematodes. During this period the ewes and lambs had access to commercially formulated sheep nuts and chopped Lucerne hay ad libitum. All animals were faecal sampled periodically to ensure no parasitic nematodes were present. Four female lambs per selection line were chosen and at an average age of 84 days (standard deviation, 6.8 days) these animals were sacrificed and duodenum tissue promptly collected. The tissue was frozen in liquid nitrogen and stored at -80°C. No adult nematodes could be detected in the abomasums or intestines of the animals confirming their naïve status. The average live weight of the lambs pre-slaughter was 23.4 kg (standard deviation, 3.8 kg). No significant differences were observed between the weights of the animals in the two lines at any stage. All procedures were approved by the AgResearch Invermay Animal Ethics Committee, formally constituted under the New Zealand Animal Welfare Act.

### RNA preparation

Total RNA was isolated from the duodenum of each animal using TRIzol (Invitrogen) and was cleaned using an RNeasy kit (Qiagen). RNA integrity was confirmed by denaturing agarose gel electrophoresis and RNA was quantitated using a NanoDrop^® ^ND-1000 Spectrophotomoter (NanoDrop Technologies). First-strand cDNA was made from 25 μg of total RNA by anchored oligo(dT)_20_-primed reverse transcription incorporating amino-modified dNTPs, and was subsequently labelled indirectly by fluorescent coupling of Cy™3 and Cy™5 mono-reactive dyes (Amersham) using the SuperScript™ Indirect cDNA Labeling System (Invitrogen) according to the manufacturer's instructions.

### Array preparation

Ovine cDNA libraries were prepared from 27 tissues by MWG Biotech (Germany). These libraries were single-pass sequenced from the 5' end generating expressed sequence tags (ESTs). The inserts, representing ovine expressed sequences, were then amplified in 96 well plates using the universal primers SP6 and T7 in 50 μl reactions (1.5 mM MgCl_2_, 2 mM each dNTP, 45 pmol primers and 2.5 units AB Red Hot *Taq *polymerase). The reactions were denatured at 94°C for 3 minutes then cycled 36 times at 94°C for 45 sec, 55°C for 45 sec and 72°C for 60 sec. Finally products were extended at 72°C for 5 min. The PCR products were verified by agarose gel electrophoresis, precipitated and resuspended in printing solution as described previously [25]. At the University of Otago Genomics Facility an ovine microarray consisting of 19,968 spots was printed onto poly-L-lysine coated glass slides using an ESI array robot with 32 split pinheads depositing 0.6 nl with a 100 μm spot size. After printing the slides were UV irradiated to cross-link the DNA to the polylysine coating.

### Slide hybridisation, scanning and normalisation

Slides were prehybridised by incubation for 45 min at 42°C in 50 ml of 5 X SSC, 0.1% SDS, 1% BSA. They were then rinsed twice in deionised water, once in isopropanol and dried. The labelled cDNA was denatured by heating at 95°C and then combined with 50 μl SlideHyb #1 (Ambion) and applied to slides. Slides were hybridised at 54°C for 16 hours in humidified chambers (Monterey). Post-hybridisation the slides were washed in the dark at 54°C for 10 min in 2 X SSC, 0.1% SDS, 5 min in 1 X SSC and 10 min in 0.1 X SSC. All buffers were filtered through 0.22 μm filters. The slides were dried and scanned in a ScanArray 5000 (Packard Biosciences) and the dual images collected in TIFF format. The combination and processing of the images was carried out using GenePix Pro (Axon Instruments). The data for each slide was normalised following the procedure of Baird *et al*., [71]. For each EST on the array, the normalised data from all 16 slides was combined and a number of average statistics calculated. ESTs were excluded from further analysis if they had more than 6/16 bad spots. The remaining ESTs were sorted based on the modified T value [72] for the log ratio of the mean [Additional file 3]. ESTs where the tail of the probability plot was more extreme than the 95% confidence limit [73] were counted as differentially expressed (Figure 1). All the microarray information has been submitted into the National Centre for Biotechnology Information (NCBI) Gene Expression Omnibus (GEO) website [74]. The accession number for the experiment series is GSE3738.

### Northern blotting

20 μg of total RNA from duodenum tissue was separated on a 1% formaldehyde agarose gel and transferred to Hybond nylon membrane (Amersham) by capillary transfer. Probes were generated by amplification from the original EST using the gene specific primers listed in Table 5. The PCR products were verified by sequencing, labelled with α^32^P-dCTP (Amersham) using the RadPrime DNA labelling system (Invitrogen) and unincorporated radioactivity removed using the High Pure PCR product purification kit (Roche). Membranes were prehybridised for 45 min at 42°C in 10 ml ULTRAhyb (Ambion). The probes were diluted 1:10 in 10 mM EDTA and denatured by boiling for 10 min followed by cooling on ice for 5 min. After addition of 0.5 ml of ULTRAhyb, the probe was added to the prehyb solution and incubated overnight at 42°C. The membranes were then washed twice for 5 min at room temperature in 2 X SSC, 0.1% SDS followed by 2 X 15 min in 0.1 X SSC, 0.1% SDS at 42°C. The membranes were then exposed to BioMax XAR film (Kodak). The images were scanned using an ImageScanner (Amersham) and the resulting images quantitated using ImageQuant TL (Amersham) and normalised to *GAPDH *mRNA levels. This gene was not differentially expressed in the microarray experiment making it a suitable choice of housekeeping gene for normalisation.

### Microarray data interpretation

All ESTs in the ovine libraries, along with all ovine ESTs deposited in NCBI were assembled into contigs using CAP3 [75] after an initial clustering step using BLAST. ESTs on the array were annotated by finding the human RefSeq (RefSeq release as at 11/4/2005) corresponding to the contig to which they belonged using BLASTN [76] and the following options: -e 0.01 -v 5 -b 5. Each EST was annotated with the top human RefSeq hit. In the case of the differentially expressed ESTs, a more stringent E value cutoff of 1 × 10^-18 ^was applied. In cases where the EST matched more than one transcript variant of a gene then the top hit is listed. All analysis hereafter refers to the annotated RefSeq genes. Gene Ontology (GO) terms significantly associated with the differentially expressed genes were found using the Expression Analysis Systematic Explorer (EASE). The list of differentially expressed genes was submitted to EASEonline [77]. The background list submitted included all human RefSeqs on the array. EASE calculates overrepresented functional gene categories compared to all the genes on the array [31].

A region of 1,500 bp upstream of the transcription start site of each of the RefSeq genes on the array was retrieved from the human genome browser at University of California-Santa Cruz [78] and sequences were masked for repeats through the retrieval process. The choice of promoter length was based on the report that approximately 75% of human core promoters lie within 1,500 bp of the transcription start site [79]. Of the 9,238 unique RefSeqs on the array, promoter regions for 8,809 RefSeqs were retrieved. Motifs in the promoter regions of the differentially expressed RefSeq genes were detected using the MEME motif detection program [32]. The motif lengths ranged between 6 and 12 bases, any number of repetitions of the motif was permitted, the reverse complement was allowed and the top 15 motifs were identified in each group The identified motifs were also compared to the transcription factor database, TRANSFAC release 8.1, [33] using the method described in Aerts *et al*., [80]. Each motif was permuted 1,000 times and best matches and their level of significance are reported. The full list of motifs identified in the promoter regions of the genes more highly expressed in susceptible and resistant animals, and their top TRANSFAC hits are available in Additional files 1 and 2 respectively. In order to carry out a MAST analysis [81] motifs with a Pearson's correlation coefficient greater than 0.6 were identified and excluded from further analysis. Promoter regions of both groups of differentially expressed genes and all RefSeq genes on the array were subsequently screened for the presence of all unique motifs using MAST. Once again, reverse complement orientation of the motifs was allowed and the results obtained were analysed using Fisher's exact test based on the number of RefSeq promoters with a combined motif significance of P < 0.0001 in each of the groups.

## Authors' contributions

OMK extracted all RNA, carried out the microarray experiments, Northern blotting and Gene Ontology analysis as well as preparing the manuscript. AZ carried out the MEME and MAST analysis. TW and DLH designed, constructed and validated the ovine cDNA arrays. GJG was responsible for all animal care, handling and experimentation. DBB developed the microarray normalisation procedure and carried out statistical analysis, AFM curated the EST database, assembled the contigs and provided bioinformatics support. AMC and JCM conceived the study, participated it its design, coordination, analysis and writing. All authors read and approved the final manuscript.

## Supplementary Material

Additional File 1 This file contains all motifs detected by MEME in the promoter regions of genes more highly expressed in susceptible animals. Click here for file

Additional File 2 This file contains all motifs detected by MEME in the promoter regions of genes more highly expressed in resistant animals. Click here for file

Additional File 3 This file ranks the ESTs on the array according to their differential expression and contains statistical information for each EST on the array. Click here for file

## Figures and Tables

**Table 1:** Genes more highly expressed in the duodenum of genetically susceptible animals compared to resistant

**Human Refseq: Genbank ** **accession number**	**Gene description**	**Gene name**	**Ensembl human gene ** **Cytogenetic band**	**Contig BLAST ** **E value**	**Fold increase ** **in expression**	**P value**
NM_003226*	Intestinal trefoil factor 3	*TFF3*	21q22.3	3 × 10^-59^	1.2	7.7 × 10^-20^
NM_006398*	Ubiquitin D	*UBD*	6p22.1	1 × 10^-52^	1.3	1.8 × 10^-12^
NM_003122*	Serine protease inhibitor, Kazal type 1	*SPINK1*	5q32	2 × 10^-58^	1.3	5.5 × 10^-10^
NM_001009555	SH3 domain protein D19	*EVE1*	4q31.3	0	1.8	3 × 10^-9^
NM_004295	TNF receptor-associated factor 4	*TRAF4*	17q11.2	0	1.1	1.3 × 10^-8^
NM_022342	Kinesin family member 9	*KIF9*	3p21.31	0	1.4	1.8 × 10^-7^
NM_005646	TAR (HIV) RNA binding protein 1	*TARBP1*	1q42.2	0	1.2	2 × 10^-7^
NM_033503	Bcl2 modifying factor	*BMF*	15q15.1	1 × 10^-18^	1.2	3.7 × 10^-7^
NM_003392	Wingless-type MMTV integration site family, member 5A	*WNT5A*	3p14.3	6 × 10^-58^	1.2	4.1 × 10^-7^
NM_000179	MutS homologue 6	*MSH6*	2p16.3	0	1.2	8 × 10^-7^
NM_001655	Archain 1	*ARCN1*	11q23.3	0	1.2	1.4 × 10^-6^
NM_079423	Myosin light polypeptide 6	*MYL6*	12q13.2	0	1.1	1.9 × 10^-6^
NM_006184	Nucleobindin 1	*NUCB1*	19q13.33	0	1.1	3.4 × 10^-6^
NM_178156	Fucosyltransferase 8	*FUT8*	14q23.3	0	1.3	4.5 × 10^-6^
NM_005533	Interferon induced protein 35	*IFI35*	17q21.31	3 × 10^-77^	1.1	6.7 × 10^-6^
NM_000062	Serine (or cysteine) proteinase inhibitor, clade G	*SERPING1*	11q12.1	2 × 10^-50^	1.2	9 × 10^-6^
NM_002133	Decycling heme oxygenase 1	*HMOX1*	22q12.3	0	1.1	1.1 × 10^-5^
NM_002816	Proteasome 26S subunit	*PSMD12*	17q24.2	0	1.2	1.1 × 10^-5^
NM_002116	Major histocompatibility complex, class I, A	*HLA-A*	6p21.33	8 × 10^-35^	1.1	1.2 × 10^-5^
NM_013442	Stomatin-like 2	*STOML2*	9p13.3	0	1.2	1.2 × 10^-5^
NM_201265	Bone marrow stromal cell-derived ubiquitin-like 7	*BMSC-UbP*	15q24.1	0	1.3	1.5 × 10^-5^
NM_016248	A kinase anchor protein 11	*AKAP11*	13q14.11	1 × 10^-44^	1.2	1.8 × 10^-5^
NM_006808	Sec61 beta subunit	*SEC61B*	9q22.33	1 × 10^-138^	1.1	1.9 × 10^-5^
NM_032492	Jagunal homologue 1	*JAGN1*	3p25.3	1 × 10^-180^	1.1	2.8 × 10^-5^
NM_002083	Glutathione peroxidase 2	*GPX2*	14q23.3	0	1.2	3.3 × 10^-5^

* More than one EST corresponding to this gene was differentially expressed and their probabilities were combined.

**Table 2:** Genes more highly expressed in the duodenum of genetically resistant animals compared to susceptible

**Human RefSeq: Genbank ** **accession number**	**Gene description**	**Gene name**	**Ensembl human gene ** **cytogenetic band**	**Contig BLAST ** **E value**	**Fold increase** ** in expression**	**P value**
NM_003789	TNFRSF1A-associated via death domain	*TRADD*	16q22.1	1 × 10^-136^	1.3	2.9 × 10^-8^
NM_000988	Ribosomal protein L27	*RPL27*	17q21.31	4 × 10^-98^	1.2	5.9 × 10^-7^
NM_007209	Ribosomal protein L35	*RPL35*	9q33.3	1 × 10^-141^	1.2	2.4 × 10^-6^
NM_014716	Centaurin, beta 1	*CENTB1*	17p13.1	7 × 10^-62^	1.3	2.9 × 10^-6^
NM_000477	Albumin	*ALB*	4q13.3	1 × 10^-151^	1.2	3.4 × 10^-6^
NM_175571	IMAP family member 8, GTPase	*GIMAP8*	7q36.1	2 × 10^-22^	1.2	4.2 × 10^-6^
NM_006118	HS1 binding protein	*HAX1*	1q21.3	0	1.2	4.6 × 10^-6^
NM_005801	Putative translation initiation factor	*SUI1*	17q21.2	0	1.1	4.7 × 10^-6^
NM_182752	Family with sequence similarity 79, member A	*FAM79A*	1p36.32	1 × 10^-117^	1.2	5.3 × 10^-6^
NM_006827	Transmembrane trafficking protein	*TMP21*	14q24.3	4 × 10^-19^	1.1	7.7 × 10^-6^
NM_001107	Erythrocyte acylphosphatase 1	*ACYP1*	14q24.3	1 × 10^-114^	1.2	9.2 × 10^-6^
NM_000211	Integrin, beta 2	*ITGB2*	21q22.3	0	1.2	1 × 10^-5^
NM_000613	Hemopexin	*HPX*	11p15.4	1 × 10^-121^	1.2	1.5 × 10^-5^
NM_145869	Annexin A11	*ANXA11*	10q22.3	1 × 10^-122^	1.2	2 × 10^-5^
NM_002872	Small GTP binding rho family protein	*RAC2*	22q13.1	0	1.2	2 × 10^-5^
NM_033657	Death associated protein 3	*DAP3*	1q22	0	1.1	2.5 × 10^-5^

**Table 3:** GO terms significantly associated with the differentially expressed genes

	GO term	No. of RefSeqs associated with term	Fisher exact score
**Susceptible**	Response to biotic stimulus	6	0.00012
	Response to stimulus	7	0.00028
	Response to external stimulus	6	0.00067
	Defense response	5	0.00072
	Response to stress	5	0.00124
	Organismal physiological process	5	0.00425
	Immune response	4	0.00486
	Response to pest/pathogen/parasite	3	0.00956
	Cytoskeleton	3	0.0397
**Resistant**			
	Cellular Process	9	0.0426

**Table 4:** MAST results

	Resistant (16)	Susceptible (25)	Array (8809)	Fisher exact score
Proportion of genes with significant combination of resistant motifs	0.50	0.00	0.05	4.7 × 10^-7^
Proportion of genes with significant combination of susceptible motifs	0.06	0.44	0.08	4.6 × 10^-6^

Proportion of promoter regions which contain the combination of motifs detected by MEME with P < 0.0001. Numbers in parenthesis represent the total number of genes in each group. In both cases the combination of motifs is found more frequently in the group in which it was originally identified. The significance of this is given by Fisher's exact score.

**Table 5:** Primers used for Northern blot probe amplification

Primer Name	Primer Sequence 5'-3'
GAPDHF	TGAAGGTCCGTGTGAACGGATTTGGC
GAPDHR	CATGTAGGCCATGAGGTCCACCAC
GIMAP8F	TGCATACCTTTCCCTCTTCG
GIMAP8R	GCCTAGCCGTAAATAGGAACC
SPINK1F	CGGTGCAGTTTTCAACTGAG
SPINK1R	CCAAGCACGCATTGTAGTGT
TFF3F	TACGGTCCGGATTCCGGG
TFF3R	CCTCATGCTGAGCACGGG
