# Supplementary material for: Gene expression profiling of Naïve sheep genetically resistant and susceptible to gastrointestinal nematodes
Source: BMC Genomics. 2006 Mar 6;7:42. doi: 10.1186/1471-2164-7-42 (PMC1450279; doi:10.1186/1471-2164-7-42)
Supplement: Additional File 1 — This file contains all motifs detected by MEME in the promoter regions of genes more highly expressed in susceptible animals. [file 1471-2164-7-42-S1.pdf]

Additional file 1: All motifs detected in the promoter regions of genes more highly expressed in susceptible animals

Motif (length)	E value	Sequence Logo	Bit score	No. of sites	TRANSFAC hit	Description	Score	P value
1 (12)	3.9×10^{-6}	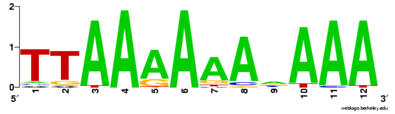	16.7	25	BSAP	B cell lineage specific activator	3.1086	0.156
2 (8)	1.2×10^{-2}	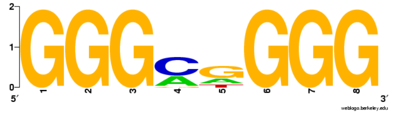	13.3	50	PPARG	peroxisome proliferative activated receptor, gamma	3.5056	0.03
3 (12)	2.3×10^{3}	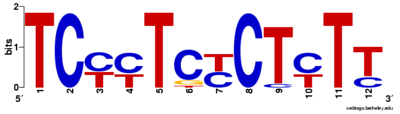	18.0	15	DR3	nuclear receptor subfamily 1, group I	2.8787	0.054
4 (12)	1.4×10^{5}	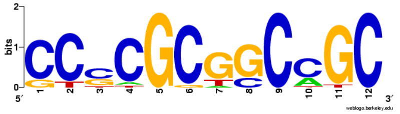	17.1	17	EGR	early growth factor	2.6570	0.121
5 (12)	2.0×10^{7}	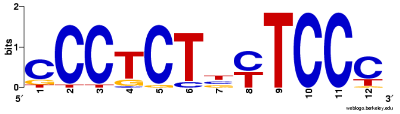	16.7	17	PAX5	paired box gene 5	2.2633	0.034
6 (12)	2.0×10^{7}	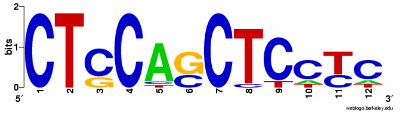	17.2	14	PAX5	paired box gene 5	2.4121	0.053
7 (8)	1.4×10^{11}	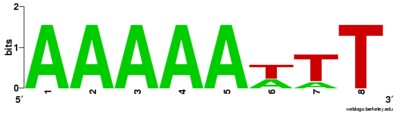	14.4	5	CIZ	Cas-interacting zinc finger protein	1.6467	0.006
8 (8)	4.2×10^{10}	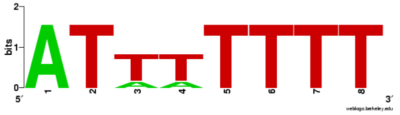	14.6	5	PR	progesterone receptor	3.5862	0.102

Motif (length)	E value	Sequence Logo	Bit score	No. of sites	TRANSFAC hit	Description	Score	P value
9 (6)	7.1×10^{11}	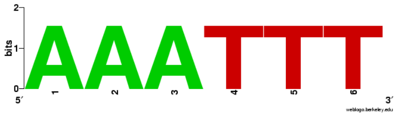	12.1	5	OCT1	Octamer-binding transcription factor-1	1.2166	0.00
10 (12)	1.3×10^{11}	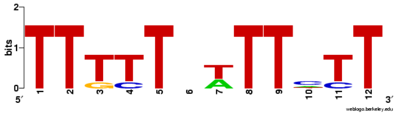	18.1	5	HNF1	transcription factor 1, hepatic	2.0877	0.003
11 (6)	5.8×10^{12}	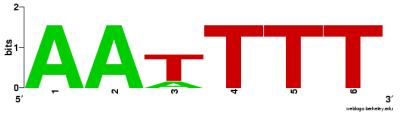	11.3	5	PAX5	paired box gene 5	3.3418	0.054
12 (6)	7.1×10^{11}	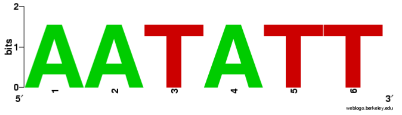	12.1	5	OCT1	Octamer-binding transcription factor-1	1.0262	0.00
13 (6)	2.0×10^{13}	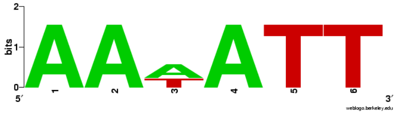	11.1	5	PR	progesterone receptor	3.4683	0.024
14 (6)	7.1×10^{11}	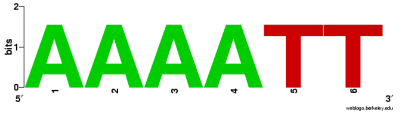	12.1	5	PR	progesterone receptor	3.6993	0.00
15 (11)	1.4×10^{12}	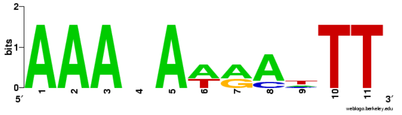	16.5	5	MEF2	myocyte enhancer factor 2	1.7762	0.06
