# Supplementary material for: Gene expression profiling of Naïve sheep genetically resistant and susceptible to gastrointestinal nematodes
Source: BMC Genomics. 2006 Mar 6;7:42. doi: 10.1186/1471-2164-7-42 (PMC1450279; doi:10.1186/1471-2164-7-42)
Supplement: Additional File 2 — This file contains all motifs detected by MEME in the promoter regions of genes more highly expressed in resistant animals. [file 1471-2164-7-42-S2.pdf]

Additional file 2: All motifs detected in the promoter regions of genes more highly expressed in resistant animals

Motif (length)	E value	Logo	Bit score	No. of sites	TRANSFAC hit	Description	Score	P value
1 (12)	4.7	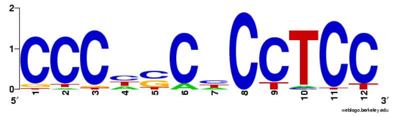	15.1	27	SRF	serum response element	2.0915	0.063
2 (11)	7.3 x 10^{5}	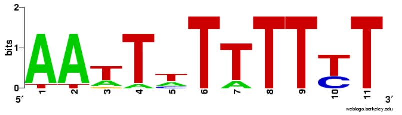	16.8	11	PAX5	paired box gene 5	3.3317	0.007
3 (12)	2.4 x 10^{5}	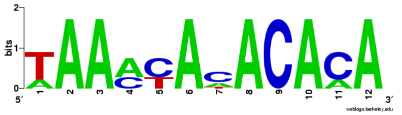	19.4	8	HFH4	forkhead box J1	1.6232	0.009
4 (11)	6.1 x 10^{5}	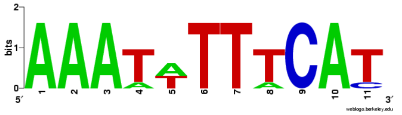	19.4	6	PAX5	paired box gene 5	3.1281	0.009
5 (11)	4.9 x 10^{6}	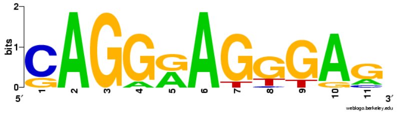	15.5	17	EGR	early growth factor	2.1310	0.000
6 (10)	2.7 x 10^{6}	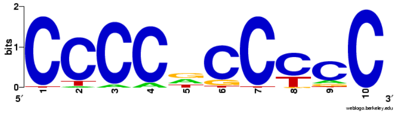	13.1	36	SP1	Sp1 transcription factor	0.6101	0.038
7 (12)	3.3 x 10^{6}	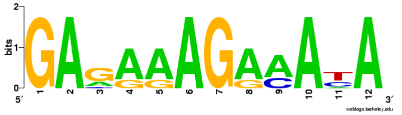	18.4	9	IRF	muscle specific ring finger protein 1	2.4637	0.155
8 (12)	3.3 x 10^{6}	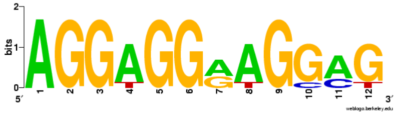	19.7	7	PAX9	paired box gene 9	2.7644	0.04

Motif (length)	E value	Logo	Bit score	No. of sites	TRANSFAC hit	Description	Score	P value
9 (12)	1.3 x 10^{9}	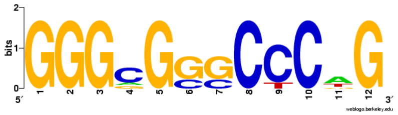	18.7	7	HOX13	homeo box B13	3.2295	0.048
10 (12)	3.4 x 10^{10}	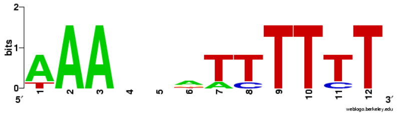	17.1	5	STAT5A	signal transducer and activator of transcription 5A	2.1485	0.001
11 (6)	1.3 x 10^{13}	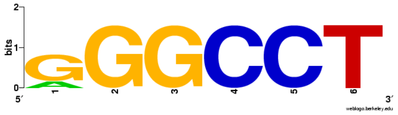	11.1	5	CAAT	CCAAT box	3.0462	0.214
12 (6)	1.6 x 10^{11}	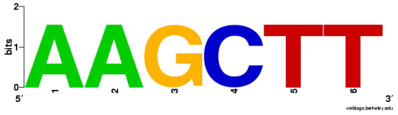	12.1	5	CAAT	CCAAT box	3.0722	0.069
13 (8)	1.9 x 10^{13}	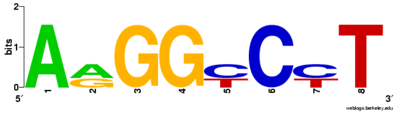	13.0	5	HNF4	transcription factor 1, hepatic	2.3839	0.226
14 (6)	3.4 x 10^{11}	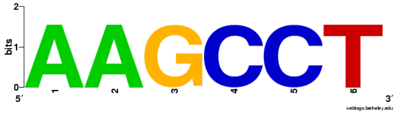	12.0	5	TAACC	TATA box	2.2700	0.120
15 (8)	2.8 x 10^{14}	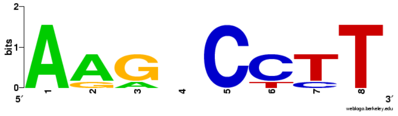	11.2	5	HNF4ALPHA	hepatocyte nuclear factor 4, alpha	1.6697	0.098
